# Genotoxicity and mutagenicity assessment of a standardized extract (P2Et) obtained from *Caesalpinia spinosa*

**DOI:** 10.1016/j.toxrep.2020.12.024

**Published:** 2020-12-26

**Authors:** R. Ballesteros-Ramírez, M.I. Durán, S. Fiorentino

**Affiliations:** Grupo de Inmunobiología y Biología Celular, Facultad de Ciencias, Pontificia Universidad Javeriana, Bogotá, Colombia

**Keywords:** *C. spinosa*, *Caesalpinia spinosa*, P2Et, *Caesalpinia spinosa extract*, PERK, protein kinase R like endoplasmic reticulum kinase, XBP1, X-box binding protein 1, ICD, immunogenic cancer cell death, CAM, complementary alternative therapies, *Caesalpinia spinose* extract, P2Et, Genotoxicity, Ames test, Micronucleus test, Herbal drug, Antitumoral, Polyphenols

## Abstract

The standardized P2Et extract obtained from *Caesalpinia spinosa* has shown antioxidant, and direct antitumor activity, but also activation of specific immune response through the induction of tumor immunogenic cell death in breast and melanoma cancer models. The present work evaluated the mutagenicity and genotoxicity profile of P2Et to continue the development of the P2Et. Genotoxicity was evaluated by OECD 1997 a guideline and mutagenicity by OECD 2016. At P2Et’s doses of 500, 1000, and 2000 mg/kg body weight in mice (*Mus musculus*), the difference between the number of micronuclei in PCE of the groups were not statistically significant (17 (negative control), 15 (500 mg/kg), 15 (1000 mg/kg), 19 (2000 mg/kg) and 271 (positive control). Similarly, P2Et did not induce gene mutations by base pair changes or frameshifts in the genome of *Salmonella Typhimurium* strains TA98, TA100, TA102, TA1535 and TA1537 at the tested range of concentrations up to 5000 μg/plate in the absence and presence of metabolic activation. Therefore, the P2Et was considered as non- mutagenic and non-genotoxic at the conditions of the tests.

## Highlights

• The P2Et extract was assessed for genotoxicity and mutagenicity activity.
• The P2Et extract showed no genotoxicity in Micronucleus assay.
• The P2Et extract showed no mutagenicity in Ames test.

## Graphical abstract

**Figure:**
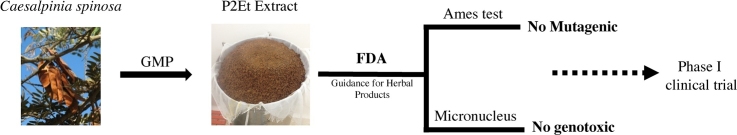

## 1 Introduction

Up to four billion people living in the developing world are estimated to depend on herbal medicines as the primary source of medical care and traditional medical practice. The use of herbs is considered an integral part of the culture in those communities. In the developed world, the use of herbal remedies has also been widely accepted and is becoming mainstream in the UK and the rest of Europe, as well as in North America and Australia [1]. Although herbal medications are better tolerated than chemically synthesized medications, potentially serious adverse events, including herb-drug interactions, have been described [2]. Among the herbal compounds used, polyphenol-type antioxidants report benefits in the prevention and treatment of diseases with a metabolic and/or inflammatory component [3]. Despite these benefits, certain polyphenols may have carcinogenic/genotoxic/effects, interfere with thyroid hormone biosynthesis and may induce hepatotoxicity and bleeding lessons in the gastrointestinal tract [[4], [5], [6], [7]]. Other adverse effects including gastrointestinal disorders, insomnia, raised blood pressure and skin/subcutaneous reactions are reported [34].

*Caesalpinia spinosa* has been traditionally used by Colombian indigenous located in Caribbean coast. *Caesalpinia spinosa* is a shrub commonly called Dividivi or Tara, with a pantropical distribution in forests, savannas and semi-deserts. Dividivi fruits have 40–60 % of Hidrolizable tannins with gallic acid as the main constituent. Tannic acids, present in Dividivi, inhibits the growth of tumors induced by chemical agents [8], and the carcinogenesis induced by UV light in mice [9] Also, gallic acid shows anti- oxidant, anti-allergenic, anti-mutagenic, anti-carcinogenic and anti-inflammatory properties [10] and decreased proliferation of cervical cancer cells, leukemia and melanoma [11,12].

In our group, an extract derived from *Caesalpinia spinosa* called P2Et was obtained, which was chemically normalized and is now produced under GMP in LABFARVE Labs. Multiple esters derived from gallic acid have been reported, such as ethyl gallate, methyl gallate, ethyl 4,5-digaloyl quinate, methyl 4,5-digaloyl quinate, ethyl 3,5-digaloyl quinate, 4,5- digaloylquinic, ethyl 3,4,5-trigaloyl quinate, ethyl 5-galloyl quinate, among others in this extract [14].

The P2Et extract has been identified as antioxidant and more recently antitumor and immunomodulatory [14,[16], [17], [18]]. In C57BL/6 mice, treatment with the P2Et extract performed twice per week during 21 days, increased the number of CD8+ lymphocytes and the frequency of activated CD4+ and CD8+ lymphocytes and also the concentration of some serum cytokines (IL-10, IL-17, IFN-alpha, IL-6, IL-4 and IL-2) showing the immunomodulatory effects of P2Et [13]. In cancer models, the activity of the P2Et extract appears to be specific depending on the tumor cell, since mitochondrial depolarization is observed only on the K562 and 4T1 cell lines, but not on the MCF-7 human breast cancer line. Although in the latter, it shows a clear adjuvant activity when extract was combined with doxorubicin, vincristine, camptothecin or taxol, increasing the sensitivity to these drugs at least by four times [17]. We also reported a decrease of primary tumor growth and metastatic foci in spleen, liver, and lung after *in vivo* treatment of immunocompetent mice orthotopically transplanted with 4T1 (highly metastatic breast cancer cell line) or B16-F10 (melanoma cell line) [15,16,18]. P2Et induces a substantial selective induction of specific ER-stress mediators in B16-F10 melanoma cells, dependent of PERK, and independent of Xbp1. P2Et-driven activation of PERK in melanoma cells promotes ER-calcium release, disrupts mitochondrial membrane potential, and triggers upregulation of ICD drivers as surface calreticulin expression, and extracellular release of ATP and HMGB1 [18,19,15].

There is a lack of mutagenicity and genotoxicity studies that identify if some chromosomal aberrations or genetic alterations might be induced by polyphenol-enriched extracts without isolating any compound. These tests are necessary to ensure the safety of herbal pharmaceutical preparations made from extracts. Preclinical mutagenicity and genotoxicity studies are a fundamental requirement to access the clinical stages of safety and efficacy in humans with herbal drugs like P2Et. Therefore, mutagenicity and genotoxicity studies were performed to demonstrate the absence of chromosomal aberrations or genetic alterations induced by the P2Et extract obtained from *C. spinosa*.

## 2 Material and methods

The project was approved by the Ethics Committee of the Faculty of Sciences of the Pontificia Universidad Javeriana.

The genotoxicity and mutagenicity tests were performed in TECAM in Brazil following the guidelines defined by the OECD [22,23]; the facilities were certified in GLP at the date of execution of the studies. Animals were maintained in the test facilities according to local and international requirements, based on the Guide for the Care and Use of Laboratory Animals [24]. Animals showing continuing signs of severe distress and/or pain at any stage of the tests were euthanized. Procedures for animal care and criteria for making the decision to euthanize an animal were based on the Guidance Document on the Recognition, Assessment and Use of Clinical Signs as Humane Endpoints for Experimental Animals Used in Safety Evaluation [25].

### 2.1 P2Et extract

Pods of *Caesalpinia spinosa* (Feuillée ex Molina) Kuntze (Dividivi or tara) were collected in Villa de Leyva, Boyacá, Colombia and identified by Carlos Alberto Parra of the National Herbarium of Colombia (copy number of voucher COL 588448), permission for Scientific Research in Biological Diversity No. 5 of May 10, 2011, and Contract for Access to Genetic Resources and/or Derivative Products for Scientific Research without Commercial Interest No. 220 of 2018.

Briefly, fresh pods from *C. spinosa* were dried under airflow in a solar oven at 35°C and ground down to obtain plant material dried. This plant material was certified according to FDA-Guidelines for herbal medicinal products. Subsequently, the plant material was extracted with ethanol (96%) in a recirculating percolator. The ethanol crude extract was concentrated under vacuum, trapped on silica gel, and excess humidity removed at 25°C. Afterward, the ethanol extract was fractionated with ethyl acetate. The P2Et standardized extract was obtained in LABFARVE laboratories in GMP conditions. This preparation is manufactured in a validated production process according to GMP. The P2Et is a homogeneous and loose brown powder. The extract was certified according to the FDA-Guidelines for herbal medicinal products.

**Figure 1:**
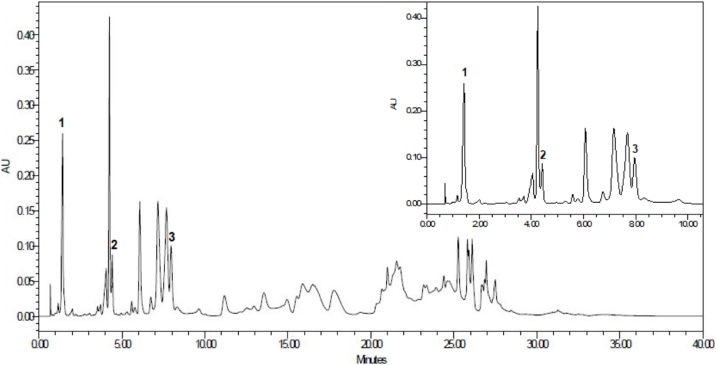
UPLC-PDA chromatogram at 274 nm of P2Et extract. Peak Identification: (1), Gallic acid; (2), Methyl gallate; (3), Ethyl Gallate.

For the standardization, multiple compounds derived from gallic acid were identified such as ethyl gallate, methyl gallate, ethyl 4,5-digaloyl quinate, methyl 4,5-digaloyl quinate, ethyl 3,5-digaloyl quinate, 4,5-digaloylquinic, ethyl 3,4,5-trigaloyl quinate, ethyl 5-galloyl quinate, among others. Three main compounds were used for the normalization of P2Et: Ethyl gallate, methyl gallate and gallic acid. The percentage of the three main compounds is as follows: methyl gallate (10.40%), ethyl gallate (18.16%) and gallic acid (30.74%). With these specifications and the standardized production, it is guarantee high batch-to-batch consistency for P2Et extract [14]. The chromatographic profile is presented in Fig. 1.

The batch used for the study was the IV. The three compounds utilized for the normalization were in the range for this batch.

### 2.2 Mammalian erythrocyte micronucleus test

#### 2.2.1 Animals and housing conditions

The animals (Species*: Mus musculus,* Strain: Swiss mice) were purchased from an external supplier (Anilab, Paulínia - SP) and were submitted to a complete clinical evaluation, including neurological and gastrointestinal evaluation, skin evaluation for ectoparasites, observation of body lesions, bleeding and/or nasal or oral secretions, dehydration and prostration. Only animals with good health were used in the present study; the animals were not vaccinated or treated with drugs during the acclimation and testing periods. Animals were randomly selected from a pool of received animals, with 9 weeks old and weight within the established. The animals were 9 weeks old at starting the test.

The mice were kept in a specific room with an average temperature of 24.3 °C, average relative humidity of 72.1 % and photoperiod 12 h of light and 12 h of darkness.

Pelleted commercial diet for rodents (Purina Labdiet 5010®) was provided *ad libitum* throughout acclimatization and test periods, except prior to dosing. Food was analyzed periodically for microbiological contaminants. The food and the water were autoclaved before giving them to the mice. Food was analyzed for microbiological and chemical contaminants. Filtered water was provided ad libitum throughout acclimatization and test periods. The drinking water was analyzed for chemical and microbiological contaminants.

#### 2.2.2 Preparation of P2Et and route of administration

The P2Et was diluted in demineralized water at constant volume of 5 mL/kg body weight, and administered orally (gavage). The dilutions were prepared freshly before administration, and after recording the weight of each animal for calculating the volume to be administered.

#### 2.2.3 Tolerability test and dose selection

In the tolerability test, doses were set considering two treatments (0 h and 24 h). To establish the MTD fixed doses were selected according to Mackay & Elliott [26] [26]. Three animals per sex were treated with 320 mg/kg body weight, considering the central doses (range: 5–2000 mg/kg body weight) and lack of information about the toxicity of the extract.

#### 2.2.4 Treatment schedule

Animals were treated twice at 0 and 24 h (two treatments at 24 h interval) and sampled once between 18 and 24 h following the final treatment. Only males were used since the lack of substantial difference in toxicity between sexes was demonstrated in the tolerability test (Section 2.2.3). Positive and negative controls were included using the same treatment schedule as the test substance.

**Figure 2:**
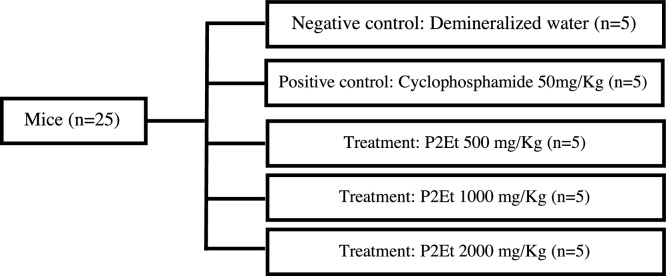
Treatment schedule and groups in the *Mammalian Erythrocyte Micronucleus Test*.

Five animals were included into the following treatment groups, negative control (demineralized water), positive control (cyclophosphamide at a dose of 50 mg/kg body weight), P2Et (500 mg/kg body weight), P2Et (1000 mg/kg body weight), and P2Et (2000 mg/kg body weight). The positive control was chosen according to the OECD 474 “Mammalian Erythrocyte Micronucleus Test” (Fig. 2).

#### 2.2.5 Slides preparation and analysis

Immediately following euthanasia (18–24 h after the final treatment), femurs were dissected from each animal. The bone marrow was flushed out by gentle flushing and aspiration with fetal calf serum. The cell suspension was centrifuged (centrifuge brand Macro IV EV025) at 1000 rpm for 5 min and the supernatant was discarded. The pellet was re-suspended in fetal calf serum and a drop of the resuspended cell pellet was spread on to clean glass slides and air-dried. Slides were air-dried, stained in Wright-Giemsa (Laborclin® and Merck, respectively), and coded with the same numbers as used in animal identification.

Slides were blind evaluated using an optical microscope (1000×, oil immersion). The number of polychromatic erythrocytes, normochromatic erythrocytes and the ratio of polychromatic erythrocytes for total erythrocytes were established for each animal by scoring a total of 500 erythrocytes (PCE + NCE = TE). The normal values were established in the laboratory considering the negative historical control data. For each animal, the number of micronucleated polychromatic erythrocytes was counted in 4000 PCE.

### 2.3 Bacterial reverse mutation test (Ames Test)

Five strains of *Salmonella typhimurium* acquired from Moltox® (Boone, NC, USA) were used TA 98, TA100, TA102, TA1535 and TA1537 in the presence and absence of the post-mitochondrial fraction (S9 fraction) prepared from livers of Sprague-Dawley male rats treated with enzyme inducing agent (Aroclor 1254) and acquired from Moltox® (Boone, NC, USA) was used.

#### 2.3.1 Genotype confirmation test

The genotypes of the strains were checked in order to ensure the originals genetic characteristics of bacteria through growth on selective media. It was analyzed their sensitivity to UV-radiation and crystal violet, resistance to ampicillin and tetracycline were checked. The strains used yield spontaneous revertants within the frequency ranges expected described for the test.

#### 2.3.2 Positive control substances

The chemicals showed in Table 1 were used as positive control substances.

**Table 1:** Compounds utilized in the Ames Test with and without metabolic activation.

Metabolic Activation	Strain	Compound	Vehicle	Concentration
Absent	TA98	2-Nitrofluorene	DMSO	2.0 μg/plate
	TA100	Sodium azide	DMSO	5.0 μg/plate
	TA102	Mitomycin C	Deionized water	0.5 μg/plate
	TA1535	Sodium azide	DMSO	5.0 μg/plate
	TA1537	ICR 191-Acridine	Deionized water	1.0 μg/plate
Present	All	2-aminoantracene	DMSO	2.5 μg/plate

#### 2.3.3 Metabolic activation system (S9 Fraction and S9 Mix)

Post-mitochondrial fraction (S9 fraction) prepared from livers of Sprague-Dawley male rats treated with enzyme inducing agent (Aroclor 1254) and acquired from Moltox ® (Boone, NC, USA) was used.

The S9 mix was freshly prepared on the day of the test according to Maron and Ames [27] containing the following components per mL: Deionized water (385 μL), 0.2 M phosphate buffer pH 7.4 (500 μL), 1 M glucose-6-phosphate solution (5 μL), 0.1 M NADP solution (40 μL), 0.4 M MgCl2.6H2O solution + 1.65 M KCl solution (20 μL) and S9 fraction (50 μL). During the experiment the S9 mix was placed in an ice bath.

#### 2.3.4 Preliminary cytotoxicity range finding

A range-finding test by direct plate incorporation using only the strain TA100 was performed in the absence of metabolic activation to determine the upper limits of concentrations, cytotoxicity and solubility of P2Et and to select concentrations for the mutagenicity test, TA100 contains the R-factor plasmid (pKM101), which increases chemical and spontaneous mutagenesis by enhancing the error-prone DNA repair system present in the strains, that is why is used as a standard tester strain and recommended by the ICH(R2). The following concentrations were used: 15, 50, 150, 500, 1500 and 5000 μg/plate. All plating was done in triplicate, except for positive controls, for which it was done in two replicates.

For the direct plate incorporation test without metabolic activation, 0.1 mL of the P2Et, 0.1 mL of a fresh bacterial culture grown overnight (18 h) and 0.5 mL of buffer were added to 2.0 mL of top agar. The contents of each tube were mixed and poured over the surface of a minimal agar plate. After solidification the plates were incubated for 72 ± 3 h at 37 ± 1 °C.

#### 2.3.5 Direct plate incorporation test – mutagenicity test

As no inhibition of the bacterial background was observed in the range-finding test, the same concentrations of the test substance were analyzed with adequate intervals between test points using a maximum of 5000 μg/plate. All plating was done in triplicate, except for the positive controls (for which it was done in two replicates).

The test in the absence of metabolic activation was performed as described in the range finding test. For test with metabolic activation, 0.1 mL of P2Et, 0.1 of a fresh bacterial culture grown overnight (18 h) and 0.5 mL of S9 mix were added to 2.0 mL of top agar. The contents of each tube were mixed and poured over the surface of a minimal agar plate. After solidification the plates were incubated for 72 ± 3 h at 37 ± 1 °C.

#### 2.3.6 Evaluation of the assay

Results were presented as number of revertant colonies per plate and concentration and by the mutation rate, which corresponds to the ratio between the number of revertants induced by the test substance and number of revertants observed in the negative vehicle control. The number of revertants observed in the vehicle control of each strain was compared to the expected range reported in the literature and to the historical control values.

### 2.4 Statistical analysis

The statistical package SPSS, version 23 was used for data analysis. For micronucleus test, a modified chi-squared test (χ²) was employed. The results obtained in positive and negative control were compared to establish the validity of the test. For the final evaluation of the test substance for genotoxic potential, the results obtained were considered as to the biological relevance, and the statistical analysis as a base. The results for the P2Et were compared with the negative control only if the micronuclei showed a bigger number than the negative control. For the test substance (P2Et) to be considered clearly positive one of the treatment groups should present a statistically significant increase in the frequency of micronucleated immature erythrocytes compared to the negative control and this increase must be dose dependent.

In the Ames assay, a test substance is considered to be active in the test system if the mutation rates after 72 hours of incubation are higher than or equal to 2 for strains TA98, TA100 and TA102 or higher than or equal to 3 for strains TA1535 and TA1537 (OECD 1997a, Maron and Ames, 1983). To confirm a positive result the analysis of variance of the data set should indicate significant results (*p* ANOVA< 0.05) and a clear dose-related increase in the number of revertants should be observed. A test substance for which the results do not meet the above criteria is considered non-mutagenic. The analysis of variance (*p* ANOVA) indicates statistically significant differences among the average number of revertants in different concentrations of test substance. When *p* ANOVA value is less than 0.05 it is concluded that there is a significant difference in at least one of the concentrations. The dose-response effect is evaluated by means of simple linear regression, where the model Revertants (dose) = intercept + slope.dose is adjusted by least-squares method. The main indicators used in this analysis are the slope and its descriptive level (*p*-value). When *p*-value <0.05, it is concluded that there is a variation between the number of revertants as a function of product concentration.

## 3 Results

### 3.1 Mammalian erythrocyte micronucleus test (*Mus musculus*)

#### 3.1.1 Tolerability test and dose selection

In tolerability test of P2Et were tested doses of 320 mg/kg body weight and 2000 mg/kg body weight. After administration of the test substance, the animals did not show clinical signs of toxicity. The next dose to be tested was 2000 mg/kg body weight. This dose was considered as the MTD to be used in the definitive test. Other doses selected in the study were 1000 mg/kg. body weight (MTD/2) and 500 mg/kg body weight (MTD/4). No differences in toxicity between sexes was demonstrated in the tolerability test.

#### 3.1.2 Definitive test

A total of 20,000 PCE were analyzed per group, the number of micronuclei counted in each group were as follow: 17 (negative control), 15 (500 mg/kg), 15 (1000 mg/kg), 19 (2000 mg/kg) and 271 (positive control). The difference between the number of micronuclei in PCE of the groups treated with 2000 mg/kg body weight and the negative control group were not statistically significant (χ² = 0.11; p = 0.739). The experimental groups treated with 500 mg/kg body weight and 1000 mg/kg body weight showed less number of micronuclei than the negative control, so no statistics was performed for this group (Table 2).

**Table 2:** *In vivo* mouse bone marrow micronucleus assay conducted with P2Et.

Treatment	BW Initial (g)*	BW Final (g)*	PCE/TE*	Total MNPCE **	MNPCE*	% MNPCE	*p* values
Demineralized Water (5 mL/kg bw)	31.4 ± 3.2	31.7 ± 2.7	0.60 ± 0.05	17	3.4 ± 0.89	0.09 ± 0.02	NA
Cyclophosphamide (50 mg/kg bw)	33.2 ± 1.4	32.9 ± 1.9	0.22 ± 0.06	271	54.2 ± 7.56	1.36 ± 0.19	<0.001
P2Et (500 mg/kg bw)	33.0 ± 2.6	32.0 ± 2.0	0.57 ± 0.05	15	3.0 ± 0.71	0.08 ± 0.02	NA
P2Et (1000 mg/kg bw)	31.8 ± 1.9	30.8 ± 2.5	0.58 ± 0.05	15	3.0 ± 1.0	0.08 ± 0.03	NA
P2Et (2000 mg/kg bw)	30.3 ± 1.0	30.0 ± 1.5	0.59 ± 0.05	19	3.8 ± 0.84	0.10 ± 0.02	0.739

PCE: polychromatic erythrocyte; TE: total erythrocytes; MNPCE: micronucleated polychromatic erythrocyte; bw: body weight: NA: No applied. * Data expressed as mean and standard deviation. ** Data expressed as total of MNPCE in the experimental group.

The sensitivity of the test was confirmed by a significant increase in micronuclei when compared positive and negative control groups (χ² = 224.01, p < 0.001).

In the analysis of the slides of the group tested with the highest test dose (2000 mg / kg bw), the ratio of PE to total erythrocytes was 59 %. The frequency of polychromatic erythrocytes (PE) in total erythrocytes (ET) did not show a decrease of 20 % in relation to the current negative control (60 %) indicating test substance cytotoxicity in the bone marrow. The low toxicity of the test substance is expected based on the preliminary test with the maximum dose (2000 mg/kg bw) and absence of clinical signs of toxicity during the study, for this reason plasma analysis was not performed.

### 3.2 Bacterial reverse mutation (Ames Test)

The counts recorded on negative control plates showed appropriate results with all tester strains. The positive control chemicals clearly induced marked increases in revertant colony numbers with all the bacterial strains, confirming the sensitivity of cultures and activity of S9 mix (Table 3).

**Table 3:** Results of P2Et tested with *Salmonella typhimurium* strains TA98, TA100, TA102, TA1535 y TA1537 (mutagenicity test; with and without metabolic activation).

Substance	Dose (μg/plate)	Revertants per plate									
		TA 98		TA 100		TA 102		TA 1535		TA 1537	
		−S9	+S9	−S9	+S9	−S9	+S9	−S9	+S9	−S9	+S9
P2Et	0^a^	37 ± 6	26 ± 4	109 ± 8	102 ± 6	281 ± 19	290 ± 21	18 ± 3	22 ± 1	9 ± 2	9 ± 1
	15	30 ± 4	28 ± 2	113 ± 7	133 ± 15	295 ± 11	292 ± 22	20 ± 4	20 ± 3	10 ± 3	12 ± 1
	50	35 ± 3	28 ± 2	105 ± 10	112 ± 12	284 ± 7	277 ± 12	21 ± 4	19 ± 1	8 ± 1	11 ± 1
	150	33 ± 8	25 ± 6	97 ± 7	107 ± 9	288 ± 23	281 ± 18	16 ± 1	21 ± 2	10 ± 2	11 ± 2
	500	26 ± 5	27 ± 4	92 ± 13	95 ± 5	266 ± 6	292 ± 7	18 ± 2	18 ± 1	9 ± 1	11 ± 3
	1500	31 ± 3	27 ± 6	105 ± 5	99 ± 11	314 ± 4	276 ± 33	18 ± 4	17 ± 4	10 ± 1	10 ± 1
	5000	30 ± 4	26 ± 5	124 ± 6	96 ± 8	270 ± 13	269 ± 2	17 ± 3	18 ± 2	10 ± 2	10 ± 1
	p ANOVA	0.248	0.966	0.008*	0.005*	0.011*	0.671	0.426	0.201	0.590	0.222
	p value	0.350	0.759	0..010*	0.060	0.405	0.128	0.377	0.084	0.479	0.494

2-Nitrofluorene	2.0	140 ± 11	-	-	-	-	-	-	-	-	-
2-Aminoanthracene	2.5	-	143 ± 7	-	461 ± 45	-	913 ± 13	-	361 ± 79	-	117 ± 12
Sodium Azide	5.0	-	-	805 ± 96	-	-	-	735 ± 54	-	-	-
Mitomycin C	0.5	-	-	-	-	860 ± 53	-	-	-	-	-
ICR 191-Acridine	1.0	-	-	-	-	-	-	-	-	402 ± 27	-

Positive controls: 2-Nitrofluorene, 2-Aminoanthracene, Sodium Azide, Mitomycin C, ICR 191-Acridine. -S9 = without metabolic activation; +S9 = with metabolic activation. Data expressed as mean ± standard deviation. * Statistically significant difference.

No substantial increase in revertant colony numbers of any of the five tester strains was observed following treatment with P2Et at any concentration level, neither in the presence nor in absence of metabolic activation. There was also no tendency of higher mutation rates with increasing concentrations in the range below the generally acknowledged border of biological significance.

The analysis of variance of the data set indicated significant difference (*p* ANOVA<0.05) for strains TA100 without and with metabolic activation (*p* = 0.008 and *p* = 0.005) and TA102 without metabolic activation (*p* = 0.011) in mutagenicity test. This statistical significance occurred because all strains described showed oscillations in number of revertants in different tested concentrations, except for strain TA100 (+S9) that showed signs of toxicity that caused a decrease in the number of revertants at the highest tested concentrations. However, the revertants per plate in these strains were smaller than the positive controls.

For TA98, TA102, and TA1535 the *p* ANOVA did not indicate significant differences between the revertants per plate at the concentrations tested. Consequently, the *p*-value was higher than 0.05, indicating that revertants per plate were not a function of the concentration.

## 4 Discussion

The usage of natural products in the world for prophylaxis and disease treatment in humans has expanded. Therefore, guaranteeing their safety is mandatory during the development process of this kind of medicinal products, even more so, when many of them are popularly used without having performed efficacy and safety tests, added to little or no monitoring by medical staff. On a regulatory level, in 2004 the FDA established the development guide for herbal medicines for the industry, updated in 2016. It establishes the botanical products development pathway that must be carried out to qualify for the registration process. The requirements simulate the stages of development of a pharmaceutical product of isolated molecules, such as the manufacturing process under BPM conditions, quality controls, preclinical and clinical trials that must demonstrate safety and efficacy.

Many plant extracts are fulfilling the preclinical regulatory phases to be brought to the market either as dietary supplements or as pharmaceuticals [28,29]. To start the regulatory requirements, the genotoxic and mutagenic evaluation of an extract obtained from *Caesalpinia spinosa* called P2Et was performed. The Micronucleus assay is used for evaluation of the potential mutagenic activity of new substances in order to classify them by mutagenic hazard, besides is highly efficient as an indicator of induced chromosomal lesions for the substances tested [35], additionally the evaluation of mixtures like the ones found in extracts, it has to be tested because is clear that mixtures can induce genotoxic effects even if each of the compounds separately does not manifest such activity in testing. Therefore, it is necessary to evaluate the genotoxicity of herbal drugs to ensure the safety of patients who take these products [36].

The genotoxicity and mutagenicity results in the Micronucleus and Ames Test, showed that the P2Et extract did not induce chromosomal aberrations, being classified as a non-mutagenic substance at the doses used. Despite the popular use of *C. spinosa*, no safety reports associated with infusion or standardized extracts were found. However, there are safety reports for the consumption of *C. spinosa* gum that were reported in 2012 for the evaluation of Galactomannans of the plant in cosmetics. In this report it was shown that the gum does not show toxic effects (acute and chronic toxicity at 90 days), as well as being non-irritating and non-teratogenic [20,21,[35], [36], [37]]. Polyphenol extracts like the Yukmijihwang-tang, an herbal formula used for kidney ailments such as pollakisuria (urination at short intervals) and edema, showed that is not genotoxic at the proper dose [33]. Likewise, proanthocyanidin polymer-rich fraction (F2) of stem bark of S. adstringens has no genotoxic activity. Individually tanic compound have not shown genotoxic and mutagenic effects and no induce any tumours when is administered to rats [20,21].

Non-regulatory preclinical studies in mice, evaluating the biological activity of P2Et, gave us to understand that it was not toxic, as well as the type of compounds that were identified. This study made it possible to confirm that it was not, and also to advance in the pre-clinical regulatory development of the phytomedicine. The regulatory pathway includes complementary studies to conduct a phase I clinical trial in humans, but these results provide the foundation and knowledge for optimizing the design of the oral toxicity and bioavailability studies.

## 5 Conclusions

The test substance P2Et, at doses 500 mg/kg bw, 1000 mg/kg bw and 2000 mg/kg bw, did not induce genotoxic effect to the bone marrow cells of mice after two oral administrations with a 24 h interval and at interval dose between 0 and 5000 μg/plate, the P2Et did not induce gene mutations by base pair changes or frameshifts in the genome of *Salmonella Typhimurium strains* TA98, TA100, TA102, TA1535 and TA1537 at the tested range of concentrations in the absence and presence of metabolic activation. These studies made possible to confirm that the P2Et was considered as non- mutagenic and non-genotoxic and also to advance in the pre-clinical regulatory development of the phytomedicine.

## Conflict of Interest

S. Fiorentino is an investor of a granted patent related to P2Et, S. Fiorentino and R. Ballesteros-Ramírez are partners of the DreemBio company who was a licensee of related patents. The rest of the authors declare no competing interests.

## CRediT authorship contribution statement

**R. Ballesteros-Ramírez:** Conceptualization, Methodology, Visualization, Formal analysis, Investigation, Writing - original draft. **M.I. Durán:** Methodology, Investigation, Formal analysis, Writing - review & editing. **S. Fiorentino:** Conceptualization, Methodology, Writing - review & editing, Supervision, Project administration, Funding acquisition.
